# Prediction of chronic thromboembolic pulmonary hypertension with standardised evaluation of initial computed tomography pulmonary angiography performed for suspected acute pulmonary embolism

**DOI:** 10.1007/s00330-021-08364-0

**Published:** 2021-12-02

**Authors:** Gudula J. A. M. Boon, Yvonne M. Ende-Verhaar, Ludo F. M. Beenen, Johan Coolen, Marion Delcroix, Marek Golebiowski, Menno V. Huisman, Albert T. A. Mairuhu, Lilian J. Meijboom, Saskia Middeldorp, Piotr Pruszczyk, Cornelis J. van Rooden, Anton Vonk Noordegraaf, Lucia J. M. Kroft, Frederikus A. Klok

**Affiliations:** 1 Department of Thrombosis and Hemostasis, Leiden University Medical Center, LUMC, (C7Q-14), Albinusdreef 2, Postbus 9600, 2300 RC Leiden, The Netherlands; 2 Department of Radiology and Nuclear Medicine, Amsterdam UMC, Location AMC, University of Amsterdam, Amsterdam, The Netherlands; 3 Department of Imaging and Pathology, KU Leuven, Leuven, Belgium; 4 Department of Pneumology, University Hospitals Leuven, Leuven, Belgium; 5 Department of Clinical Radiology, Medical University of Warsaw, Warsaw, Poland; 6 Department of Internal Medicine, Haga Teaching Hospital, The Hague, The Netherlands; 7 Department of Radiology and Nuclear Medicine, Amsterdam UMC, VU University Medical Center, Amsterdam, The Netherlands; 8 Department of Vascular Medicine, Amsterdam Cardiovascular Sciences, Amsterdam UMC, University of Amsterdam, Amsterdam, The Netherlands; 9 Department of Internal Medicine and Cardiology, Medical University of Warsaw, Warsaw, Poland; 10 Department of Radiology, Haga Teaching Hospital, Den Haag, The Netherlands; 11 Department of Pulmonology, Amsterdam Cardiovascular Sciences, Amsterdam UMC, VU University Medical Center, Amsterdam, The Netherlands; 12 Department of Radiology, Leiden University Medical Center, Leiden, The Netherlands

**Keywords:** Computed tomography angiography, Pulmonary artery, Pulmonary embolism, Pulmonary hypertension, Chronis thromboembolic pulmonary hypertension

## Abstract

**Objectives:**

Closer reading of computed tomography pulmonary angiography (CTPA) scans of patients presenting with acute pulmonary embolism (PE) may identify those at high risk of developing chronic thromboembolic pulmonary hypertension (CTEPH). We aimed to validate the predictive value of six radiological predictors that were previously proposed.

**Methods:**

Three hundred forty-one patients with acute PE were prospectively followed for development of CTEPH in six European hospitals. Index CTPAs were analysed post hoc by expert chest radiologists blinded to the final diagnosis. The accuracy of the predictors using a predefined threshold for ‘high risk’ (≥ 3 predictors) and the expert overall judgment on the presence of CTEPH were assessed.

**Results:**

CTEPH was confirmed in nine patients (2.6%) during 2-year follow-up. Any sign of chronic thrombi was already present in 74/341 patients (22%) on the index CTPA, which was associated with CTEPH (OR 7.8, 95%CI 1.9–32); 37 patients (11%) had ≥ 3 of 6 radiological predictors, of whom 4 (11%) were diagnosed with CTEPH (sensitivity 44%, 95%CI 14–79; specificity 90%, 95%CI 86–93). Expert judgment raised suspicion of CTEPH in 27 patients, which was confirmed in 8 (30%; sensitivity 89%, 95%CI 52–100; specificity 94%, 95%CI 91–97).

**Conclusions:**

The presence of ≥ 3 of 6 predefined radiological predictors was highly specific for a future CTEPH diagnosis, comparable to overall expert judgment, while the latter was associated with higher sensitivity. Dedicated CTPA reading for signs of CTEPH may therefore help in early detection of CTEPH after PE, although in our cohort this strategy would not have detected all cases.

**Key Points:**

• *Three expert chest radiologists re-assessed CTPA scans performed at the moment of acute pulmonary embolism diagnosis and observed a high prevalence of chronic thrombi and signs of pulmonary hypertension.*

• *On these index scans, the presence of* ≥ *3 of 6 predefined radiological predictors was highly specific for a future diagnosis of chronic thromboembolic pulmonary hypertension (CTEPH), comparable to overall expert judgment.*

• *Dedicated CTPA reading for signs of CTEPH may help in early detection of CTEPH after acute pulmonary embolism.*

**Supplementary Information:**

The online version contains supplementary material available at 10.1007/s00330-021-08364-0.

## Introduction

The potentially life-threatening disease chronic thromboembolic pulmonary hypertension (CTEPH) is preceded by a diagnosis of acute pulmonary embolism (PE) in 75% [1, 2]. Establishing a CTEPH diagnosis is known to be challenging as exemplified by a long diagnostic delay of up to 14 months, causing loss of quality-adjusted life years [3]. Moreover, the longest delays have been associated with worse pulmonary haemodynamics and excess mortality [4]. Importantly, in studies evaluating computed tomography pulmonary angiography (CTPA) and echocardiography at the time of PE diagnosis, concomitant signs of CTEPH have frequently been described, which may point to the presence of acute-on-chronic thromboembolic disease in these patients [5–9]. Alternatively, such findings may even indicate diagnostic misclassification since a first presentation of CTEPH may mimic an acute episode of PE. Vigilance on these early signs may therefore play an important role in earlier identification of patients with (high risk of) CTEPH, positively affecting patients’ prognosis.

**Fig. 1:**
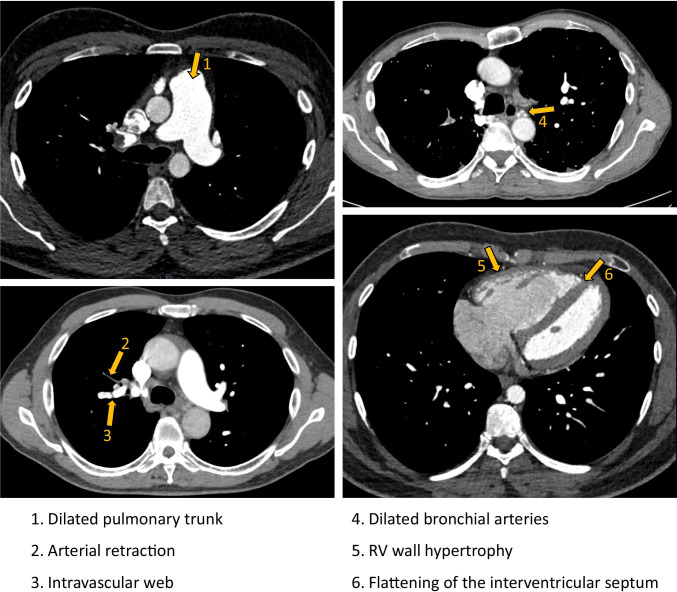
CTPA image showing the six radiological predictors of CTEPH in addition to RV/LV diameter ratio of > 1.0. Abbreviations: CTPA, computed tomography pulmonary angiography; CTEPH, chronic thromboembolic pulmonary hypertension; RV, right ventricle; LV, left ventricle

The recent InShape III study has investigated the radiological differentiation of acute PE from CTEPH on CTPAs performed for suspected PE [8]. For this study, three expert chest radiologists comprehensively assessed index CTPA scans of 50 PE patients with a subsequent CTEPH diagnosis (‘cases’) and of 50 PE patients without any signs of pulmonary hypertension (PH) on sequential echocardiograms performed > 2 years after their acute PE (‘controls’). In a standardised way, radiological signs of chronic thrombi and/or PH were scored. Multivariate analysis identified six independent, most predictive signs of a future CTEPH diagnosis (sensitivity 70%; specificity 96%; Fig. 1). Also, expert overall judgment on presence or absence of CTEPH was found to be highly predictive (sensitivity 72%; specificity 94%).

Even though a more detailed reading of index CTPA scans may help in early detection of CTEPH, several important questions need to be answered before recommending implementation in routine care [10]. Since the InShape III study included strictly selected study patients, the prevalence of the six independent predictors and the overall judgment on presence of CTEPH is unknown in unselected daily practice-based PE cohorts, as well as their prognostic value for a final CTEPH diagnosis. Thus, in the current analysis, we aimed to externally validate the predictive value of the radiological predictors proposed by the InShape III study in a larger and unselected study cohort derived from the InShape II study.

## Methods

### Study design and patients

This is a predefined post hoc analysis based on results of the InShape II study, which was a prospective, multicentre management study assessing the accuracy of a non-invasive follow-up strategy for early identification of CTEPH in consecutive patients treated for acute PE. Criteria for study inclusion have been described previously and included (1) a CTPA proven diagnosis of first or recurrent symptomatic acute PE (2) and treatment with therapeutically dosed anticoagulant therapy for at least three months according to current guidelines [11]. Main exclusion criteria were known CTEPH or PH, echocardiographically confirmed left ventricular systolic or diastolic dysfunction, or severe renal failure. The participating hospitals consisted of five academic and one teaching hospital in The Netherlands, Belgium and Poland, all of which have a dedicated expert outpatient clinic for PH care.

All study participants were managed according to the predefined InShape II algorithm, which is a screening strategy for excluding CTEPH early after acute PE (Appendix A in the Supplementary information). Firstly, the presence of high pre-test probability of CTEPH, calculated by the ‘CTEPH prediction score’, and symptoms suggestive of CTEPH were evaluated [12, 13]. Subsequently, if at least one of the ‘CTEPH rule-out criteria’ (i.e. determined by ECG and NT-proBNP, N-terminal pro-brain natriuretic peptide) was not able to preclude the presence of right ventricular (RV) pressure overload, patients were referred for transthoracic echocardiography [14, 15]. For study purposes, all patients were subjected to echocardiography after a 2-year follow-up. Diagnostic work-up of CTEPH was performed according to the 2015 ESC/ERS guidelines on PH: in case of intermediate or high echocardiographic probability of PH, patients were subjected to targeted diagnostic tests for CTEPH including ventilation/perfusion scintigraphy and right heart catheterisation (RHC) [16]. Only patients with available index CTPA scans were eligible for the current study.

The study protocol was approved by all institutional review boards of the participating hospitals and all patients provided written informed consent before the start of any study procedure.

### Objectives

The objectives of this study were (1) to examine the prevalence of the six predefined radiological predictors established in the InShape III study, as well as the overall judgment on the presence of CTEPH by expert radiologists among the InShape II study population; (2) to investigate the association between the presence of six predictors and the overall expert judgment on the presence of CTEPH with a final CTEPH diagnosis; (3) to evaluate the prognostic value of CTPA reading for a future CTEPH diagnosis in several subgroups based on pre-test probability of CTEPH and sex.

### Data collection

Of all InShape II study patients, available CTPA scans at the time of index PE diagnosis were collected post hoc at each of the six study sites. All scans had been performed using a CT scanner with at least 64 slices and a slice thickness of 1 to 3 mm. After full anonymisation and removal of meta-data, only the original axial images were available for local expert chest radiologists to re-assess the images. These radiologists all had over 15 years of experience in pulmonary CTPA reading.

All involved radiologists were unaware of the results of the InShape II screening algorithm and of the results of a 2-year follow-up, i.e. whether CTEPH was ultimately diagnosed or not. Standardised re-assessment of CTPA scans was done according to an identical scoring form as previously used in the InShape III study (Appendix B in the Supplementary information) [8]. This assessment is focussed on the presence of signs of chronicity in the morphology of the thrombi, as well as direct and indirect signs of chronic RV overload, referred to as ‘signs of PH’. The presence of an array of radiological parameters was scored including the six predetermined independent and most predictive radiological signs of a future CTEPH diagnosis: presence of intravascular webs; arterial retraction; dilatation of the bronchial arteries; dilatation of the pulmonary trunk (diameter > 30 mm or larger than aortic diameter); RV wall hypertrophy (> 4 mm); and flattened interventricular septum. Ultimately, the radiologists were asked to give an overall judgment on the presence or absence of CTEPH. If present, signs were interpreted as predictive for a future diagnosis of CTEPH since it remains unknown whether patients had CTEPH at the time of their PE diagnosis.

### Statistical analysis

Patient characteristics were described as mean with standard deviation (SD), median with interquartile range (IQR), or numbers with proportions if appropriate. Descriptive analyses were used to show the results of the CTPA reading. The number of patients judged to have chronic thrombi or PH were assessed, as well as the prevalence of the six previously mentioned predictors on the index CTPA scan. Using logistic regression analysis, both the presence of ≥ 3 of these predictors (a cut-off that was predefined in the InShape III study) and the overall judgment on the presence of CTEPH were correlated to a final CTEPH diagnosis during 2-year follow-up. Patients with high suspicion of CTEPH in whom diagnosis was not confirmed with RHC were not included in the main analysis but only in the sensitivity analysis. Measures of diagnostic accuracy were calculated with corresponding 95% confidence intervals (95%CI). All statistical tests were performed using SPSS Statistics software (version 25.0, IBM).

## Results

### Patients

Of the 424 consecutively included PE patients in the InShape II study, index CTPA scans of 341 patients were available and evaluated by six independent radiologists. The remaining 83 patients could not be included because the patients were referred for treatment to one of the study sites after the CTPA had been performed elsewhere (*n* = 68), or acute PE was diagnosed using a ventilation/perfusion scan (*n* = 15). Patients’ characteristics at baseline are presented in Table 1: mean age at the time of PE diagnosis was 56 years (SD 16) and 49% of patients were male. The index PE was a recurrent venous thromboembolism (VTE) in 21% and an unprovoked event in 55% of patients.

**Table 1:** Baseline characteristics of study participants

	Patients with available index CTPAs (*n* = 341)
Age (mean ± SD)	56 (16)
Male sex (*n*, %)	167 (49)
BMI (mean ± SD)	28 (5.9)
Unprovoked PE (*n*, %)	188 (55)
High-risk PE* (*n*, %)	9 (2.6)
A prior history of VTE (*n*, %)	71 (21)
Onset of symptoms > 2 weeks before index PE diagnosis (*n*, %)	73 (21)
*Comorbidities (n, %)*	
Anaemia	71 (21)
COPD/asthma	38 (11)
Active malignancy^#^	31 (9.1)
Diabetes mellitus	24 (7.0)
Coronary artery disease	22 (6.5)
Rheumatic disease^^^	15 (4.4)
Hypothyroidism	14 (4.1)
Known antiphospholipid antibodies	5 (1.5)
Interstitial lung disease	4 (1.2)
Inflammatory bowel disease	4 (1.2)
Major vasculitis syndromes	2 (0.6)
Prior infected pacemaker leads	1 (0.3)
Splenectomy	1 (0.3)
*Anticoagulant treatment at 3-month follow-up visit*	
DOAC	233 (68)
VKA	87 (26)
LMWH	29 (8.5)

*Note:*

^*^According to the 2019 European Society of Cardiology Guidelines on Acute PE

^*#*^Active malignancy was defined as follows: diagnosis of cancer within 6 months prior to enrolment, any treatment for cancer within the previous 6 months or recurrent metastatic cancer

^^^Rheumatic disease was defined as follows: known rheumatic arthritis, osteoarthritis, connective tissue disease, systemic lupus erythematosus, ankylosing spondylitis or Sjögren syndrome

Abbreviations: *PE*, pulmonary embolism; *SD*, standard deviation; *BMI*, body mass index; *VTE*, venous thromboembolism; *COPD*, chronic obstructive pulmonary disease; *LMWH*, low-molecular-weight heparin; *VKA*, vitamin K antagonist; *DOAC*, direct oral anticoagulant. Anaemia was defined as: males < 8.5 mmol/L or < 13.5 g/dL; females < 7.5 mmol/L or < 12.0 g/dL

CTEPH was confirmed by RHC in nine of the 341 patients (2.6%), of whom eight had been identified early by the algorithm and one during follow-up (Appendix A in the Supplementary information) [11]. In addition, CTEPH was considered ‘likely’ in three patients (0.88%) with echocardiographically determined intermediate or high probability of PH, but RHC was not performed due to severe comorbidities. In the remaining patients, CTEPH was ruled out based on the InShape II algorithm. Time between acute PE and referral for diagnostic work-up for suspected CTEPH was median 4 months (IQR 3–5).

### Prevalence of radiological signs of CTEPH

Ten CTPA scans (2.9%) had a suboptimal quality, i.e. inadequate contrast timing in six and motion artefacts in four scans, but all were of sufficient quality to be used in the analysis. Chronic thrombi were present on 74 (22%) of 341 index CTPAs (Table 2). Of the patients with chronic thrombi, 56 (76%) had no prior history of VTE. Any radiological sign of PH was reported in 104 patients (30%), and 8 of those 104 (7.7%) were ultimately diagnosed with CTEPH. The presence of either chronic thrombi or signs of PH was associated with a future CTEPH diagnosis (OR 7.8, 95%CI 1.9–32 and OR 20, 95%CI 2.4–159, respectively).

**Table 2:** Prevalence of radiological signs of chronic thrombi and PH, and of the six predefined independent predictors for a future CTEPH diagnosis after acute PE

	Total study population (*n* = 341)	CTEPH diagnosis confirmed (*n* = 9)	CTEPH ruled out (*n* = 332)
Signs of chronic thrombi present (*n*, %)	74 (22)	6 (67)	68 (20)
Signs of PH present (*n*, %) *	104 (30)	8 (89)	96 (29)
*Predefined radiological predictors of CTEPH (n, %)*			
Intravascular webs	41 (12)	5 (56)	36 (11)
Arterial retraction	41 (12)	5 (56)	36 (11)
Dilated bronchial arteries	24 (7.0)	3 (33)	21 (6)
Dilatation of the pulmonary trunk	119 (35)	7 (78)	112 (34)
RV hypertrophy	19 (5.6)	2 (22)	17 (5)
Flattening of the interventricular septum	84 (25)	3 (33)	81 (24)

*Notes:* *Concerns direct and indirect signs of chronic RV overload

Abbreviations: *CTEPH*, chronic thromboembolic pulmonary hypertension; *OR*, odds ratio; *RV*, right ventricle; *95%CI*, 95% confidence interval

### Radiological discrimination between those with and without a future CTEPH diagnosis

Of the total study population, the radiologists assigned 3 or more of the 6 predefined radiological predictors to 37 patients (11%) (Table 3). Among these 37 PE patients, 4 (11%) were ultimately diagnosed with CTEPH during follow-up, and CTEPH was ruled out in the remaining 33 (89%) patients, corresponding to an OR of 7.2 (95%CI 1.9–28). Overall, the radiologists judged CTEPH present in 27 of 341 (7.9%) patients, of whom the diagnosis was actually established in 8 (30%) and ruled out in 19 (5.7%), for an OR of 132 (95%CI 16–1109). Assessment of the presence of at least 3 predictors yielded a sensitivity of 44% (95%CI 14–79) and a specificity of 90% (95%CI 86–93), compared to 89% (95%CI 52–99.7) and 94% (95%CI 91–97) for overall judgment, respectively. The diagnostic accuracy was confirmed in the sensitivity analysis while focusing on ≥ 3 predictors (sensitivity 42%, 95%CI 15–72; specificity 90%, 95%CI 87–93) as well as the overall adjudication of CTEPH (sensitivity 75%, 95%CI 43–95; specificity 95%, 95%CI 95–97).

**Table 3:** Results of the assessment of radiological signs of CTEPH in patients ultimately diagnosed with CTEPH versus those in whom CTEPH was ruled out after 2-year follow-up

	CTEPH diagnosis confirmed, *n* = 9 (*n*, %)	CTEPH ruled out, *n* = 332 (*n*, %)	Univariate analysis (OR, 95%CI)	Sensitivity (%, 95%CI)	Specificity (%, 95%CI)	PPV (%, 95%CI)	NPV (%, 95%CI)	Positive likelihood ratio (95%CI)	Negative likelihood ratio (95%CI)
Presence of ≥ 3 of 6 predictors of CTEPH	4 (44)	33 (10)	7.2 (1.9–28)	44 (14–79)	90 (86–93)	11 (5.2–21)	98 (97–99)	4.5 (2.0–9.9)	0.62 (0.34–1.1)
Overall judgment: CTEPH present	8 (89)	19 (5.7)	132 (16–1109)	89 (52–99.7)	94 (91–97)	30 (20–41)	99.7 (98–99.9)	16 (9.5–25)	0.12 (0.0–0.8)

*Abbreviations: CTEPH*, chronic thromboembolic pulmonary hypertension; *OR*, odds ratio; *95%CI*, 95% confidence interval; *PPV*, positive predictive value; *NPV*, negative predictive value

Given the 2.6% CTEPH prevalence in our cohort, the presence of at least 3 of 6 predictors had a positive predictive value (PPV) of 11% (95%CI 5.2–21) and a negative predictive value (NPV) of 98% (95%CI 97–99), accompanied by a positive likelihood ratio (LR) of 4.5 (95%CI 2.0–9.9) and a negative LR of 0.62 (95%CI 0.34–1.1). Overall expert judgment resulted in a PPV of 30% (95%CI 20–41) and a NPV of 99.7% (98–99.9) against a positive LR of 16 (96%CI 9.5–25) and a negative LR of 0.12 (95%CI 0.0–0.8).

### Subgroup analysis

Associations between CTPA reading in the subgroups are presented in Table 4. In 96 patients (28%) with a high clinical pre-test probability based on the CTEPH prediction score (i.e. a score of > 6 points), the diagnostic accuracy and predictive value of both the assessment of radiological predictors and the overall judgment of CTEPH were roughly comparable to patients with a low pre-test probability. In the specific group of patients reporting symptoms indicative of CTEPH despite a low clinical pre-test probability, either method of CTPA reading yielded similar results to the remaining patients. There were no clear sex-related differences.

**Table 4:** Results of the assessment of CPTA reading in subgroups of the InShape II study population while completing the InShape II algorithm 3–6 months after acute PE

	Numbers (*n*, %)	Sensitivity	Specificity	PPV	NPV	Numbers (*n*, %)	Sensitivity	Specificity	PPV	NPV
Clinical pre-test probability*										
	*Low n* = *245 (n, %) including CTEPH n* = *2*					*High n* = *96 (n, %) including CTEPH n* = *7*				
Presence of ≥ 3 of 6 predictors of CTEPH	13 (5.3)	50 (1.3–99)	95 (92–97)	7.7 (1.8–27)	99.6 (98–99.9)	24 (25)	43 (9.9–82)	76 (66–85)	13 (5.3–27)	94 (90–97)
Overall judgment: CTEPH present	9 (3.7)	100 (16–100)	97 (94–99)	22 (12–37)	100	18 (19)	86 (42–99.6)	87 (78–93)	33 (21–48)	99 (93–99.7)
Presence of symptoms suggestive of CTEPH despite low clinical pre-test probability ^#^										
	*No n* = *280 (n, %) including CTEPH n* = *7*					*Yes n* = *61 (n, %) including CTEPH n* = *2*				
Presence of ≥ 3 of 6 predictors of CTEPH	34 (12)	43 (9.9–82)	89 (84–92)	8.8 (3.7–20)	98 (97–99)	3 (4.9)	50 (91.3–99)	97 (88–99.6)	33 (6.7–78)	98 (93–99.5)
Overall judgment: CTEPH present	25 (8.9)	86 (42–99.6)	93 (89–96)	24 (16–35)	99.6 (98–99.9)	2 (3.3)	100 (16–100)	100 (94–100)	100	100
Sex										
	*Male n* = *167 (n, %) including CTEPH n* = *7*					*Female n* = *174 (n, %) including CTEPH n* = *2*				
Presence of ≥ 3 of 6 predictors of CTEPH	21 (13)	43 (9.9–82)	89 (83–93)	14 (6.0–30)	97 (95–99)	16 (9.2)	50 (1.3–99)	91 (86–95)	6.3 (1.5–22)	99 (98–99.8)
Overall judgment: CTEPH present	16 (9.6)	100 (59–100)	94 (90–97)	44 (29–59)	100	11 (6.3)	50 (1.3–99)	94 (90–97)	9.1 (2.2–31)	99 (98–99.897)

*Notes:* Values of diagnostic accuracy and predictive values are denoted as percentage, 95%CI

^*^Based on a clinical CTEPH prediction score of > 6.[11; 13]

^#^ Based on a clinical CTEPH prediction score of < 7. [11; 13]

## Discussion

In this practice-based cohort of PE patients, a high prevalence of chronic thrombi (22%) and signs of PH (30%) on index CTPAs were observed by expert chest radiologists and were both associated with a future CTEPH diagnosis. Firstly, using a predefined cut-off of at least 3 of 6 radiological predictors present, 4 out of 9 cases (44%) were correctly identified. Secondly, overall expert judgment led to suspicion of CTEPH in 8 of 9 CTEPH cases (89%). Importantly, either way of CTPA reading yielded a considerably high specificity (≥ 90%) for a future CTEPH diagnosis with a positive likelihood ratio of 4.5 and 16, respectively. Subgroup analysis revealed no clear differences between patients with high versus low clinical pre-test probability, between those with or without symptoms, or between sexes.

We observed that dedicated assessment of index CTPAs for 6 specific CTPA signs performed by expert chest radiologists was highly specific for a future CTEPH diagnosis, confirming the results of the InShape III study (the derivation study) [8]. In daily practice, this means that presence of at least 3 signs on CTPAs performed for suspected PE is clinically meaningful and should prompt a high suspicion of CTEPH. Our findings also show that close CTPA reading does not identify each CTEPH case. Overall expert reading resulted in higher case finding than focusing on the previously established set of 6 objective radiological predictors only. A similar pattern of superiority was found in the InShape III study [8]. This might be explained by pattern recognition of expert radiologists, emphasising the relevance of a broad vision in predicting a future CTEPH diagnosis. Still, the radiological predictors can provide guidance in daily practice whereas such highly experienced chest radiologists often are not available.

In line with existing literature, our findings show that a careful evaluation of chronic thrombi and signs of PH is a promising approach for earlier detection of CTEPH [17–19]. In CTEPH patients, chronic thrombi at the time of acute PE diagnosis may indicate a preliminary stage of a future CTEPH diagnosis or, alternatively, denote concurrent (pre-existing) CTEPH that had not been recognised yet [20]. Although differentiation between these two is often difficult, in the current study, in only 1 of 9 cases, PH developed during long-term follow-up. Several previous studies have reported that most CTEPH patients with a history of PE already had signs of CTEPH at the time of acute PE diagnosis based on CTPA as well as echocardiography findings [5–8]. Notably, among PE patients not ultimately diagnosed with CTEPH, signs of chronicity are found in up to 20–30% of PE patients [5, 7]. The clinical relevance of prevalent chronic thrombi at the moment of acute PE diagnosis remains unknown, especially in patients without a previous episode of VTE. A recent case-cohort study analysing thrombus morphology on consecutive CTPA scans in PE and CTEPH patients revealed that webs and tapered pulmonary arteries at the time of PE diagnosis did not resolve after three months of anticoagulant treatment [21]. Moreover, these specific chronic thrombi were strongly associated with an ultimate CTEPH diagnosis, fuelling the concept of a state of chronic PE.

Refined CTPA assessment might not be sufficient as a stand-alone tool for achieving early CTEPH diagnosis. However, it may still play an important role in routine follow-up strategies among acute PE patients. In this setting, in the 2019 European Society of Cardiology Guidelines on acute PE, it was proposed to perform echocardiography in PE patients with persistent dyspnoea, functional limitations, and/or predisposing conditions for CTEPH [22]. Presence of radiological signs suggestive of CTEPH could be added to pre-test probability assessments.

Future studies should incorporate dedicated radiological evaluation in prospective validation of follow-up strategies [1, 4, 23, 24]. Notably, it has been shown that radiologists frequently miss signs of CTEPH on CTPA in clinical practice [7, 25]. Addressing this apparent lack of awareness for CTEPH is likely crucial in reducing the current diagnostic delay [10]. In this, incorporating the presence of characteristics of chronic vascular occlusions and RV overload in a structured report for each CTPA assessment will provide early guidance in differentiation between acute and chronic thrombi, which might be especially useful for radiologists without specific expertise in chest radiology [25]. Also, integration of typical CTEPH signs in artificial intelligence-based software will further improve the diagnostic accuracy of CTPA reading, but is yet subject of ongoing studies [26–29]. Strengths of our study include the prospective design, the large and practice-based population of PE patients from several European countries, and the fact that CTPA re-assessment was performed in an identical way as was done in the InShape III study, which all support the external validity of our results. Also, adjudication of a CTEPH diagnosis was done by expert PH teams. Some limitations of our study should also be acknowledged. Ideally, the sample size of this study and the number of CTEPH cases would have been larger. The InShape II study was powered on its primary endpoint, while this study was a predefined secondary outcome. Of note, this is still the largest imaging study performed in consecutive PE patients on this topic available, underlining its relevance. Also, a total of 20% of patients of the InShape II study were excluded from the current study since their CTPA scans were unavailable. Further to this, each re-evaluation was done by expert chest radiologists, but we have not determined their interobserver agreement in this study. Of note, an expert agreement was found to be good in the InShape III study. Whether we would have found comparable results when less experienced radiologists would have been asked to evaluate the CTPA scans is unclear. Lastly, 2-year follow-up echocardiography had not been performed in 4.7% of study patients, but, upon inquiry, there were no patients with any symptoms suggestive of CTEPH during follow-up.

In conclusion, dedicated assessment of the presence of signs of chronic clots or PH on CTPAs performed in the setting of suspected acute PE may contribute to earlier detection of CTEPH, validating the results of the InShape III study. Overall expert judgment yielded similar results to focussing on a predefined set of objective radiological predictors only, but performed better in terms of case finding. As stand-alone assessment, expert reading was not sufficient to identify each CTEPH patient, but would have identified the vast majority.

## Supplementary Information

Below is the link to the electronic supplementary material.

Supplementary file1 (DOCX 126 KB)

## Abbreviations

95%CI: 95% Confidence intervals
BMI: Body mass index
COPD: Chronic obstructive pulmonary disease
CTEPH: Chronic thromboembolic pulmonary hypertension
CTPA: Computed tomography pulmonary angiography
DOAC: Direct oral anticoagulant
IQR: Interquartile range
LMWH: Low-molecular-weight heparin
LV: Left ventricle
NPV: Negative predictive value
OR: Odds ratio
PE: Pulmonary embolism
PH: Pulmonary hypertension
PPV: Positive predictive value
RHC: Right heart catheterisation
RV: Right ventricle
SD: Standard deviation
VKA: Vitamin K antagonist
VTE: Venous thromboembolism

## Declarations

### Guarantor

The scientific guarantor of this publication is F.A. Klok.

### Statistics and biometry

No complex statistical methods were necessary for this paper.

### Study subjects or cohorts overlap

The current study is a predefined post hoc analysis based on results of the InShape II study, which was a prospective, multicentre management study assessing the accuracy of a non-invasive follow-up strategy for early identification of CTEPH in consecutive patients treated for acute PE. Of all 424 InShape II study patients, available CTPA scans at the time of index PE diagnosis were collected post hoc at each of the six study sites and were re-evaluated.

## References

[1] Pepke-Zaba J, Delcroix M, Lang I (2011). Chronic thromboembolic pulmonary hypertension (CTEPH): results from an international prospective registry. Circulation.

[2] Huisman MV, Barco S, Cannegieter SC (2018). Pulmonary embolism. Nat Rev Dis Primers.

[3] Boon GJAM, van den Hout WB, Barco S (2021). A model for estimating the health economic impact of earlier diagnosis of chronic thromboembolic pulmonary hypertension. ERJ Open Res.

[4] Klok FA, Barco S, Konstantinides SV et al (2018) Determinants of diagnostic delay in chronic thromboembolic pulmonary hypertension: results from the European CTEPH Registry. Eur Respir J 52(6):1801687 10.1183/13993003.01687-2018 30409820

[5] Guerin L, Couturaud F, Parent F (2014). Prevalence of chronic thromboembolic pulmonary hypertension after acute pulmonary embolism. Thromb Haemost.

[6] Lorenz G, Saeedan MB, Bullen J et al (2020) CT-based biomarkers for prediction of chronic thromboembolic pulmonary hypertension after an acute pulmonary embolic event. AJR Am J Roentgenol. 10.2214/AJR.19.22541:1-7 10.2214/AJR.19.22541 32809861

[7] Rogberg AN, Gopalan D, Westerlund E, Lindholm P (2019). Do radiologists detect chronic thromboembolic disease on computed tomography?. Acta Radiol.

[8] Ende-Verhaar YM, Meijboom LJ, Kroft LJM (2019). Usefulness of standard computed tomography pulmonary angiography performed for acute pulmonary embolism for identification of chronic thromboembolic pulmonary hypertension: results of the InShape III study. J Heart Lung Transplant.

[9] Ende-Verhaar YM, Cannegieter SC, Vonk Noordegraaf A et al (2017) Incidence of chronic thromboembolic pulmonary hypertension after acute pulmonary embolism: a contemporary view of the published literature. Eur Respir J 49:1601792 10.1183/13993003.01792-2016 28232411

[10] Delcroix M, Torbicki A, Gopalan D et al (2021) ERS statement on chronic thromboembolic pulmonary hypertension. Eur Respir J 57:2002828 10.1183/13993003.02828-2020 33334946

[11] Boon GJAM, Ende-Verhaar YM, Bavalia R (2021). Non-invasive early exclusion of chronic thromboembolic pulmonary hypertension after acute pulmonary embolism: the InShape II study. Thorax.

[12] Ende-Verhaar YMRD, Bogaard HJ, Huisman MV, Meijboom L, Vonk Noordegraaf A, Klok FA (2018). Sensitivity of a simple non-invasive screening algorithm for chronic thromboembolic pulmonary hypertension after acute pulmonary embolism. TH Open.

[13] Klok FA, Dzikowska-Diduch O, Kostrubiec M (2016). Derivation of a clinical prediction score for chronic thromboembolic pulmonary hypertension after acute pulmonary embolism. J Thromb Haemost.

[14] Klok FA, Surie S, Kempf T (2011). A simple non-invasive diagnostic algorithm for ruling out chronic thromboembolic pulmonary hypertension in patients after acute pulmonary embolism. Thromb Res.

[15] Klok FA, Tesche C, Rappold L (2015). External validation of a simple non-invasive algorithm to rule out chronic thromboembolic pulmonary hypertension after acute pulmonary embolism. Thromb Res.

[16] Galie N, Humbert M, Vachiery JL (2016). 2015 ESC/ERS guidelines for the diagnosis and treatment of pulmonary hypertension. Eur Heart J.

[17] Rajaram S, Swift AJ, Condliffe R (2015). CT features of pulmonary arterial hypertension and its major subtypes: a systematic CT evaluation of 292 patients from the ASPIRE Registry. Thorax.

[18] Grosse A, Grosse C, Lang I (2018). Evaluation of the CT imaging findings in patients newly diagnosed with chronic thromboembolic pulmonary hypertension. PLoS One.

[19] Gopalan D, Delcroix M, Held M (2017) Diagnosis of chronic thromboembolic pulmonary hypertension. Eur Respir Rev 26:160108 10.1183/16000617.0108-2016 PMC9488918 28298387

[20] Klok FA, Couturaud F, Delcroix M, Humbert M (2020) Diagnosis of chronic thromboembolic pulmonary hypertension after acute pulmonary embolism. Eur Respir J 55:2000189 10.1183/13993003.00189-2020 32184319

[21] Braams NJ, Boon GJAM, de Man FS, et al (2021) Evolution of CT findings after anticoagulant treatment for acute pulmonary embolism in patients with and without an ultimate diagnosis of CTEPH. Eur Resp J 2100699. 10.1183/13993003.00699-2021 10.1183/13993003.00699-2021 34112733

[22] Konstantinides SV, Meyer G, Becattini C (2019). 2019 ESC Guidelines for the diagnosis and management of acute pulmonary embolism developed in collaboration with the European Respiratory Society (ERS). Eur Respir J.

[23] Ende-Verhaar YM, van den Hout WB, Bogaard HJ (2018). Healthcare utilization in chronic thromboembolic pulmonary hypertension after acute pulmonary embolism. J Thromb Haemost.

[24] Tapson VF, Platt DM, Xia F (2016). Monitoring for pulmonary hypertension following pulmonary embolism: The INFORM Study. Am J Med.

[25] Boon GJAM, Jairam PM, Groot GMC (2021). Identification of chronic thromboembolic pulmonary hypertension on CTPAs performed for diagnosing acute pulmonary embolism depending on level of expertise. Eur J Intern Med.

[26] Zhai Z, Staring M, Zhou X (2019). Linking convolutional neural networks with graph convolutional networks: application in pulmonary artery-vein separation.

[27] Remy-Jardin M, Faivre J-B, Kaergel R (2020). Machine learning and deep neural network applications in the thorax: pulmonary embolism, chronic thromboembolic pulmonary hypertension, aorta, and chronic obstructive pulmonary disease. J Thorac Imaging Suppl.

[28] Jimenez-Del-Toro O, Dicente Cid Y, Platon A (2020). A lung graph model for the radiological assessment of chronic thromboembolic pulmonary hypertension in CT. Comput Biol Med.

[29] Liu W, Liu M, Guo X (2020). Evaluation of acute pulmonary embolism and clot burden on CTPA with deep learning. Eur Radiol.

