# Supplementary material for: Prediction of chronic thromboembolic pulmonary hypertension with standardised evaluation of initial computed tomography pulmonary angiography performed for suspected acute pulmonary embolism
Source: Eur Radiol. 2021 Dec 2;32(4):2178–87. doi: 10.1007/s00330-021-08364-0 (PMC8921171; doi:10.1007/s00330-021-08364-0)
Supplement: Supplementary file 1 — Supplementary file1 (DOCX 126 KB) [file 330_2021_8364_MOESM1_ESM.docx]

**Appendix A:** InShape II algorithm showing final diagnosis of CTEPH among the 341 study participants in the current study

**
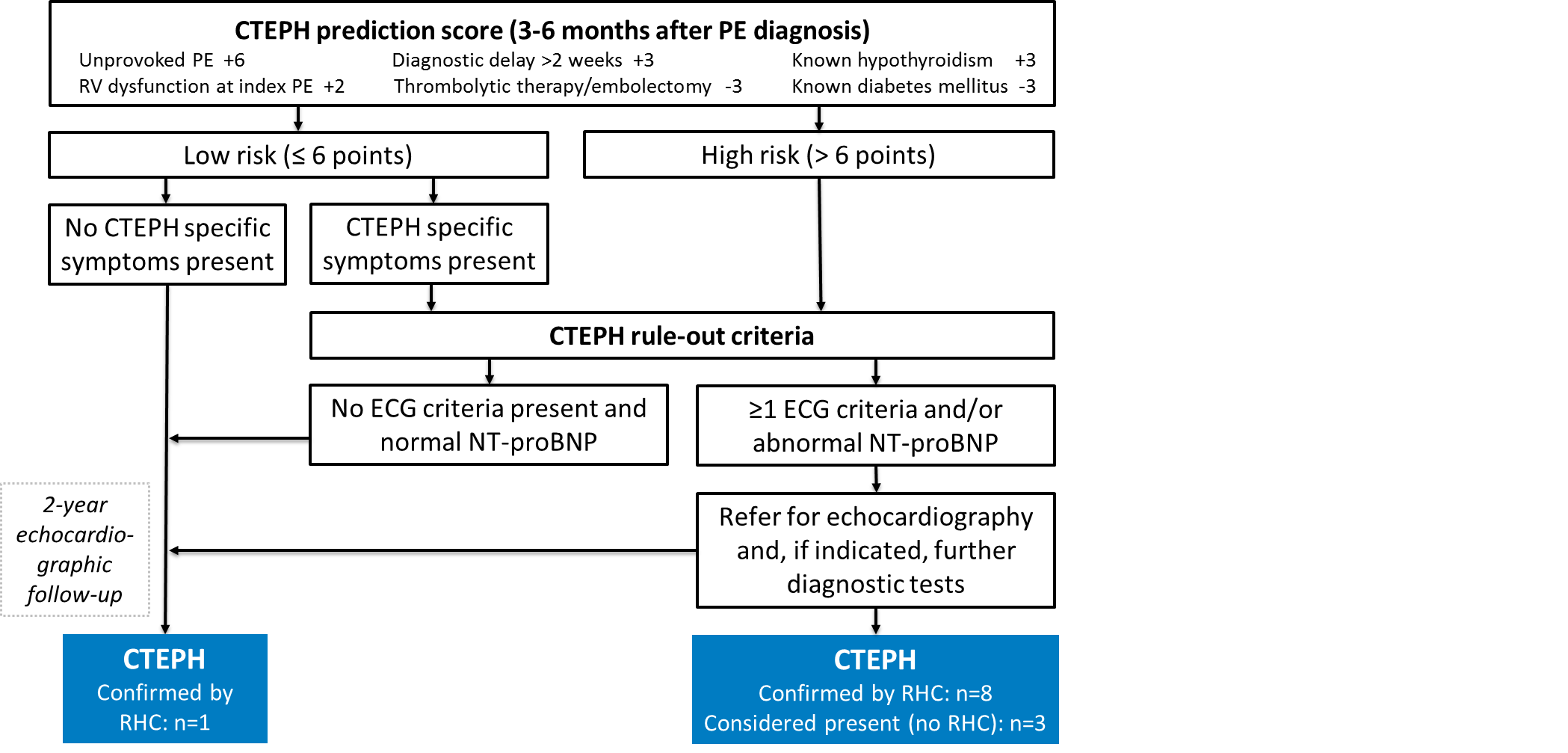
**

*Note:* The ECG criteria of RV pressure overload are: 1) rSR’ or rSr’ pattern in lead V1, 2) R:S >1 in lead V1 with R >0.5 mV and 3) QRS axis >90^o^. “Signs of PH” relate to echocardiographically determined intermediate or high probability of PH according to the 2015 ESC/ERS Guidelines for the diagnosis and treatment of PH.[13]

*Abbreviations:* PE, pulmonary embolism; CTEPH, chronic thromboembolic pulmonary hypertension; ECG, electrocardiography; NT-proBNP, N-terminal pro-brain natriuretic peptide; PH, pulmonary hypertension; RHC, right heart catheterization.

**Appendix B:** Standardized scoring form for evaluation of radiological characteristics of chronic PE and PH

| Intravascular webs/bands | | □ Yes □ No | |
| --- | --- | --- | --- |
| Thrombus attached to the vascular wall | | □ Yes □ No | |
| Complete arterial occlusion | | □ Yes □ No | |
| Arterial retraction | | □ Yes □ No | |
| Poststenotic vascular dilatation | | □ Yes □ No | |
| Mosaic perfusion | | □ Yes □ No | |
| Pulmonary infarction | | □ Yes □ No | |
| Parenchymal bands | | □ Yes □ No | |
| Pathological/dilated bronchial arteries | | □ Yes □ No | |
| RV diameter | …… mm | Dilatation | □ Yes □ No |
| RA diameter | …… / …… mm | Dilatation | □ Yes □ No |
| LV diameter | …… mm | Dilatation | □ Yes □ No |
| Pulmonary trunk diameter | …… mm | Dilatation of pulmonary trunk | □ Yes □ No |
| Aorta diameter | …… mm |  |  |
| RV wall diameter | …… mm | RV wall hypertrophy | □ Yes □ No |
| Flattening of the interventricular septum | | □ Yes □ No | |
| **Diagnosis** | | | |
| Acute PE | □ Yes □ No | | |
| Chronic PE | □ Yes □ No | | |
| PH | □ Yes □ No | | |
| CTEPH | □ Yes □ No | | |
| In case of uncertainty about diagnosis, specify why… |  | | |
| Other comments… |  | | |

*Abbreviations:* PE, Pulmonary embolism; PH, pulmonary hypertension; RV, right ventricle; RA, right atrium; LV; left ventricle; CTEPH, chronic thromboembolic pulmonary hypertension.
